# Supplementary material for: Multichannel bridges and NSC synergize to enhance axon regeneration, myelination, synaptic reconnection, and recovery after SCI
Source: NPJ Regen Med. 2024 Mar 18;9:12. doi: 10.1038/s41536-024-00356-0 (PMC10948859; doi:10.1038/s41536-024-00356-0)
Supplement: Supplementary file 1 — Supplemental Material [file 41536_2024_356_MOESM1_ESM.pdf]

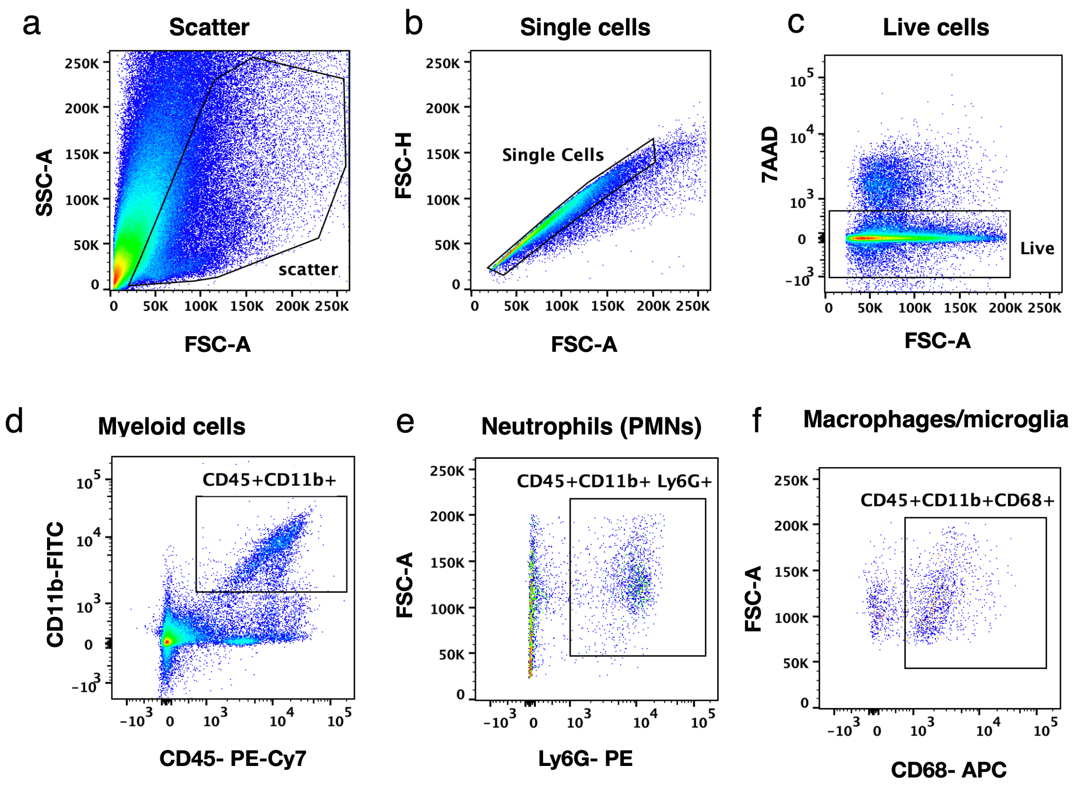

Supplemental Figure 1: Flow cytometric plots showing gating strategy for analyzing innate immune cell response in injured mouse spinal cord. (a) FSC-A vs. SSC-A scatter was used as the gate for the cell population. **(b)** FSC-A vs. FSC-H was applied to minimize doublet selection. **(c)** Selection of 7AAD negative live cells. **(d)** Live cells were gated to analyze the number of CD45+CD11b+ myeloid events. **(e-f)** Total myeloid cells were further gated to analyze the proportion of Ly6G+ PMN **(e),** and CD68+ MØ/microglia subpopulations **(f).**

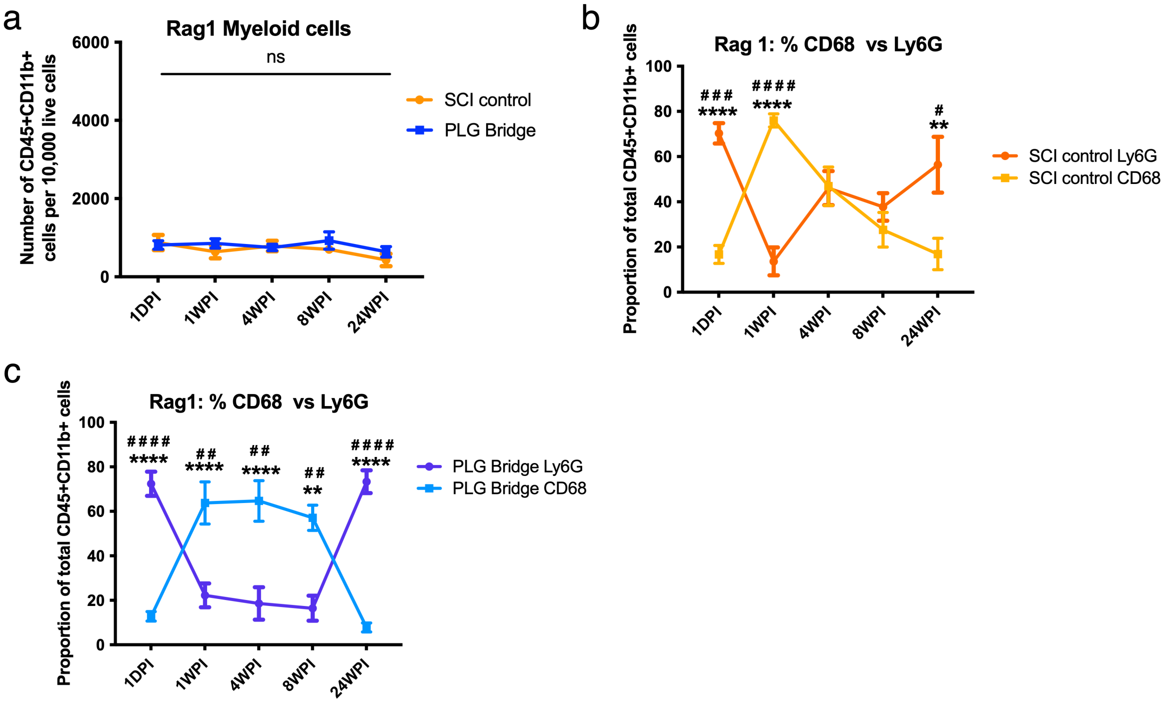

Supplemental Figure 2: Modulation of the innate immune cell response and time course by PLG bridge implantation in the injured Rag1 immunodeficient mouse spinal cord is similar to that in the injured C57Bl/6 spinal cord. (a) Total myeloid cell infiltration in SCI control (orange) groups and PLG (blue) groups over time in Rag1 mice. **(b)** Comparison of Ly6G+ PMN (dark orange circles and line) and MØ/microglia (light orange squares and line) ratios in the SCI control group of Rag1 mice. **(c)** Comparison of Ly6G+ PMN (dark blue circles and line) and MØ/microglia (light blue squares and line) ratios in the PLG group of Rag1 mice. Similar to C57BL/6 mice, the PMN population peaked acutely at 1DPI and re-emerged by 24WPI, and the MØ/microglia population was elevated over a prolonged period (up until 8 WPI) in the PLG bridge vs. SCI control group. Mean ± SEM; N=3-5 mice/group. Statistical analysis via 2-way ANOVA, followed by Sidak test (****p ≤0.0001 and **P≤0.01), or unpaired Student's t-tests at each time point ($^{####}$p ≤0.0001, $^{###}$P≤0.001, $^{##}$P≤0.01, $^{#}$ P≤0.05), as indicated.

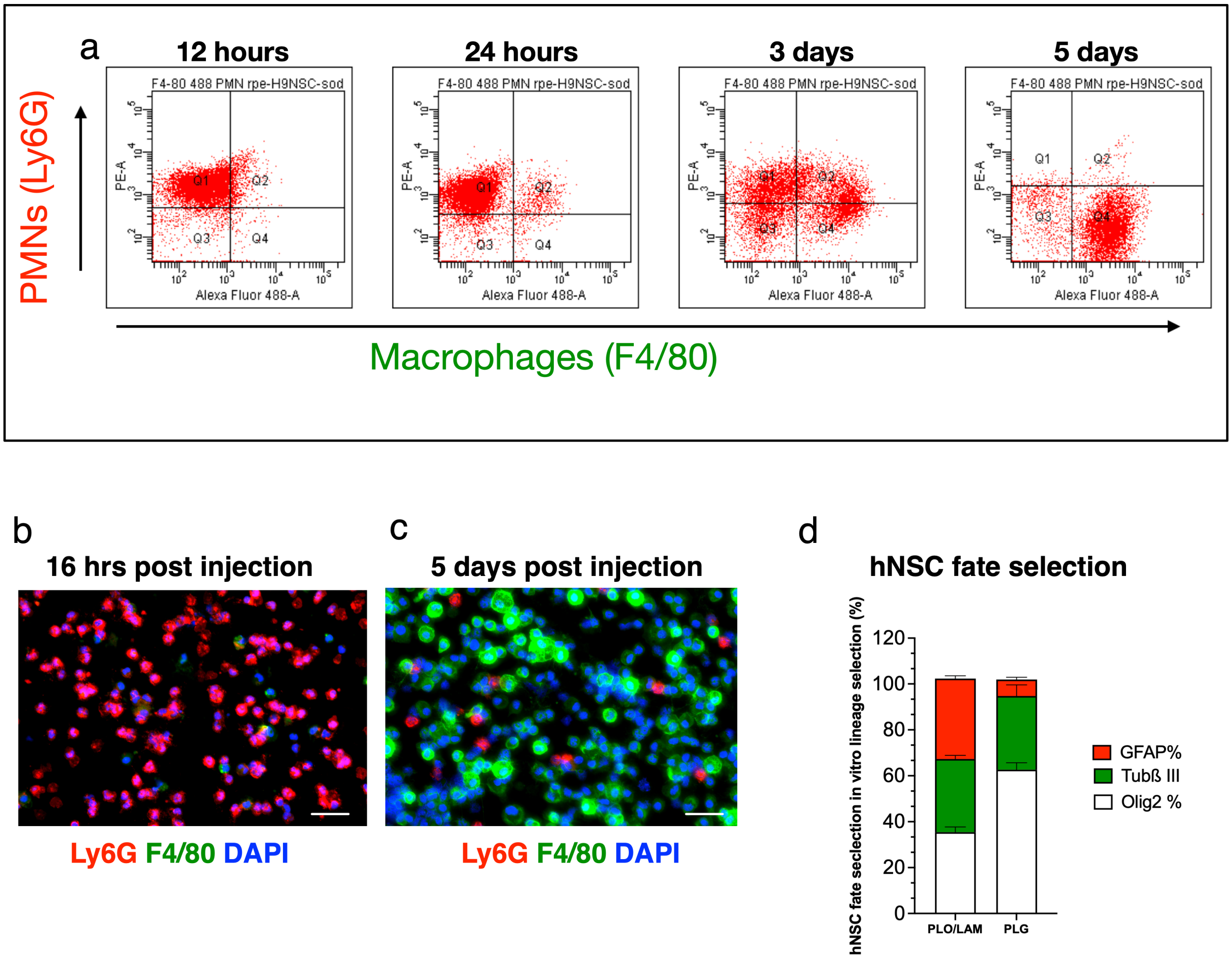

Supplemental Fig. 3: Validation of PMN and MØ CM harvest times following 12% sodium caseinate injection. (a) Peritoneal-derived PMN and MØ were isolated from immunodeficient mice after 12 hours, 24 hours, 3 days, and 5 days post 12% sodium caseinate (i.p. injection) and profiled using flow cytometry. Ly6G+ PMN are the predominant population at 12-24 hours post-injection, whereas F4/80^{+} macrophages dominate by 5 days post-sodium caseinate administration. Isolated cells were analyzed after labeling with antibodies to Ly6G (PMN) and F4/80 (macrophages). **(b-c)** Representative images of peritoneal PMN and MØ cells following flow cytometric analysis. **(b)** The Ly6G+ PMN population predominates at the time of PMN-CM harvest at 16 hours post-sodium caseinate administration. **(c)** The F4/80^{+}macrophage population predominates at the time of MØ-CM harvest at 5 days post-sodium caseinate administration. **(d)** GFAP, Tubß III, and Olig2 markers identify 100% of cells cultured on PLO/LAM (control) and PLG substrates. Scale bars: **b, c** 50μm.

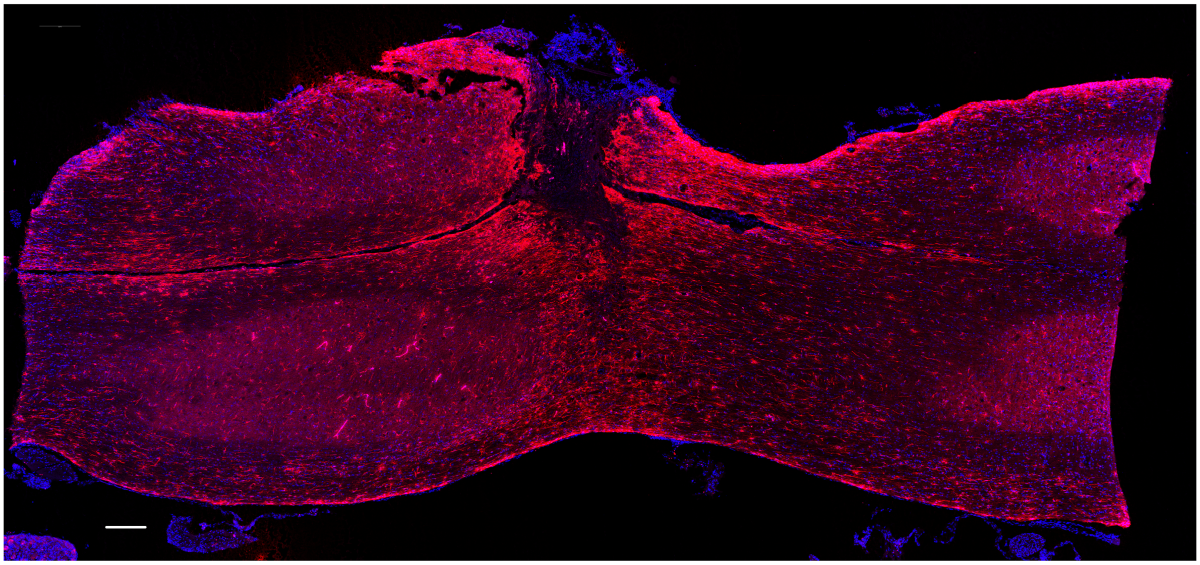

STEM121 Hoechst

Supplemental Fig. 4: hNSC distribution along the spinal cord. Representative image of a horizontal mouse spinal cord section showing the distribution of STEM121$^+$ transplanted human hNSC (red) and total cell nuclei (blue) in the PLG bridge group. Scale bars: 200μm

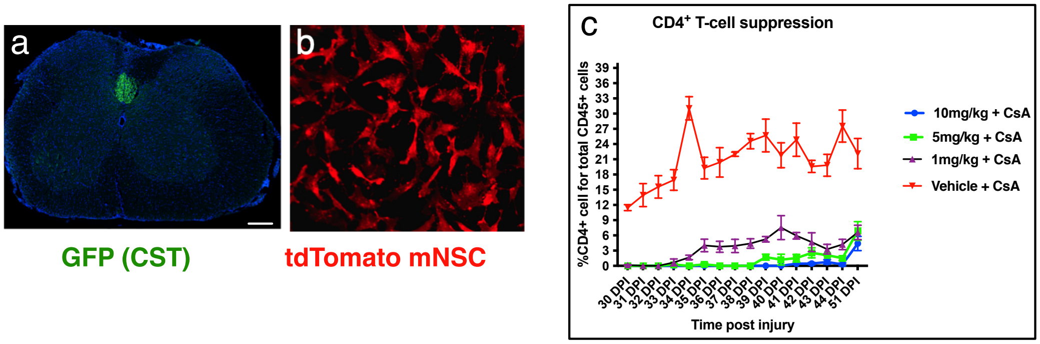

Supplemental Figure 5: CRYM-ZsGreen1 transgenic mouse reporter validation, tdTomato mNSC derivation, and anti-CD4 antibody dose optimization. (a) To investigate whether donor NSC myelinate the regenerating CST axons, we generated a CRYM-ZsGreen1 transgenic mice colony (see Methods). Consistent with our previous validation of Crym reporter expression, green fluorescence was localized selectively to CST axons in the dorsal funiculus of the spinal cord [14] (Scale bars: 200μm). **(b)** mT-mNSC were derived from embryonic day 11.5 (E11.5) cortices of tdTomato transgenic mice and cultured *in vitro* (see Methods). mNSC transplantation was conducted at passage 4 (P4). These mT-mNSC ubiquitously express membrane-targeted fluorescent tandem dimer Tomato (tdTomato), enabling the detection of transplanted mT-mNSC and myelination of host axons by these cells in the mouse-to-mouse transplantation paradigm [21]. Critically, this label allows for visualization of host axon myelination even after myelin compaction, unlike cytoplasmic labels, where the signal may be lost when myelin is compacted. CRYM-ZsGreen1 mice received a C5 hemisection SCI with immediate PLG bridge implantation and chronic mT-mNSC transplantation (30 DPI). **(c)** Mouse-to-mouse allogeneic transplantation requires pharmacological immunosuppression to enable the survival of transplanted mNSC within the host. To establish our immunosuppression protocol, we tested combined treatment with both Cyclosporin A (CsA) and anti-CD4 in C57Bl/6 mice that received a C5 hemisection SCI. CsA (10 mg/kg, s.c) injection was initiated at 28 DPI and continued daily for 21 days. A single injection of either anti-CD4 antibody (1, 5, or 10 mg/kg; i.p) or vehicle (saline, i.p.) was administered at 29 DPI. These times were selected because chronic mNSC transplantation is conducted at 30 DPI. Tail vein blood was collected and analyzed for $CD45^+/CD4^+$ cells by flow cytometry daily, beginning at 30 DPI (one day post anti-CD4 injection) for 14 days and then 7 days later at 51 DPI. CsA + Vehicle failed to suppress CD4 T-cells, however, both CsA + 5mg/kg anti-CD4 and CsA + 10mg/kg anti-CD4 effectively maintained CD4 T-cell suppression for 14 days after administration. Based on these data, our final immunosuppression regimen was as follows: CsA injection (10 mg/kg, s.c) was administered 2 days before mNSC transplantation and continued daily until sacrifice, along with 5mg/kg i.p. anti-CD4 given 1 day prior to mNSC transplantation and continued bi-weekly until sacrifice.

Supplemental Figure 6

PRV-GFP labeling in the Motor cortex and PVN of hypothalamus (2 months old mice)

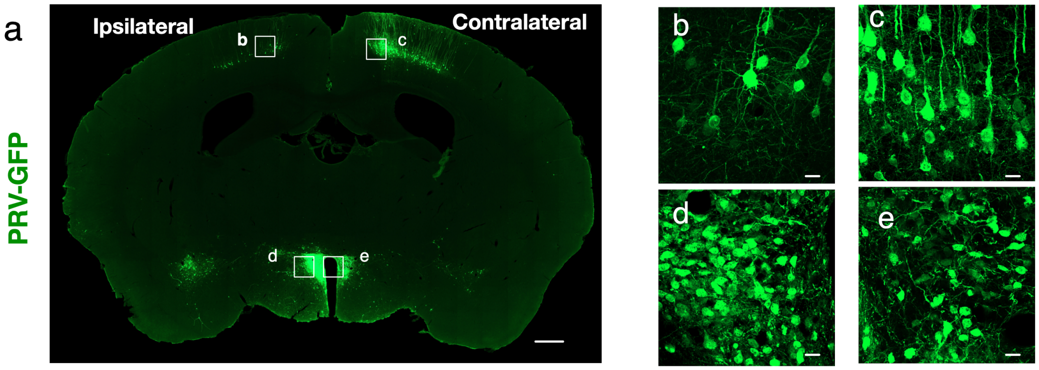

PRV-GFP labeling in the Motor cortex and PVN of hypothalamus (10 months old mice)

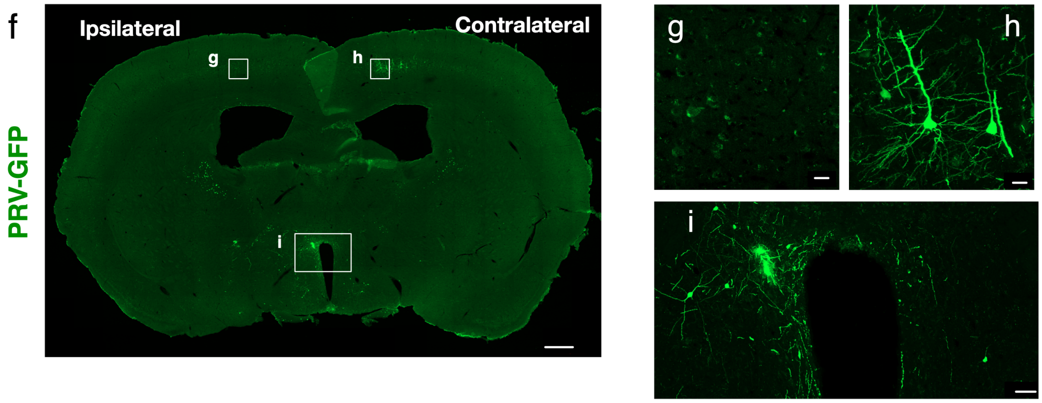

PRV-GFP labeling in naive spinal cord (10 months old)

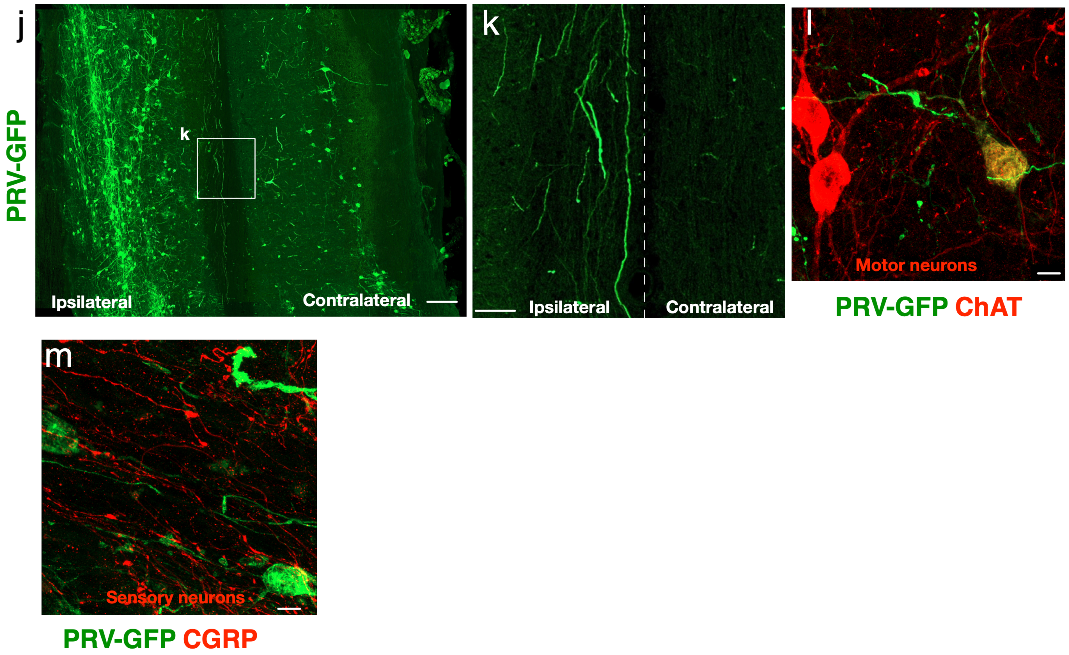

Supplemental Figure 6: Validation of PRV labeling in young and old naive mice.

(a-i) PRV was injected in the forelimb triceps of intact naive young (2 month-old) and old (10 month-old) mice and was retrogradely traced to the motor cortex and hypothalamus in the brain. **(a)** PRV retrograde tracing in 2 month-old mice. Consistent with the anatomical projection pattern of the CST, sparse PRV labeled neurons were detected in the motor cortex ipsilateral to the tricep injection **(b)**, and a majority of PRV labeled neurons were detected in the motor cortex contralateral to the tricep injection **(c).** In contrast, consistent with the anatomical projection pattern of the paraventricular nucleus (PVN) of the hypothalamus, the predominant portion of PRV labeled neurons were observed ipsilateral to the tricep injection **(d-e). (f)** Because the experimental paradigm required analysis in the chronic post-injury period, we assessed PRV labeling efficiency in 10 month-old mice. Retrograde PRV tracing was drastically reduced, possibly suggesting a weakening of synaptic connections with age. Despite the reduced number of labeled neurons detected in the brain, a similar ipsilateral/contralateral distribution pattern was apparent in the motor cortex **(g-h)** and hypothalamus **(i). (j)** PRV-GFP labeling in the spinal cord below the pyramidal decussation was predominantly ipsilateral to the tricep injection; the image shown is a merged image of multiple cervical spinal cord sections (⅙ section sampling) in the 10 month-old mice naive spinal cord. Note labeling of propriospinal neurons on the contralateral side consistent with segmental crossing fiber connections. **(k)** High magnification image showing PRV labeling of ipsilateral long tract axons. **(l)** Co-labeling with choline acetyltransferase (ChAT) demonstrates PRV labeling of primary motor neurons. **(m)** Co-labeling with calcitonin gene-related peptide (CGRP) demonstrates the absence of PRV labeling in sensory neurons. Scale bars: **a, f, j** 200μm; **b-e, g-h** 20μm; **i, k** 50μm; **l, m** 10μm.

a

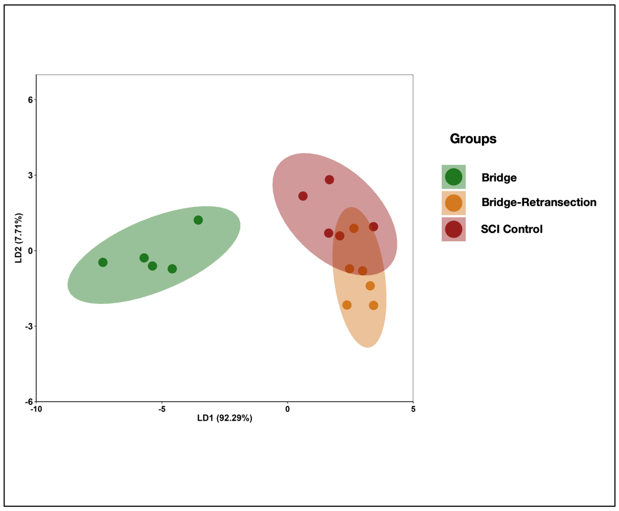

b

Catwalk Parameters	LD1	LD2
Run_Duration_(s)	-1.566	-0.758
Run_Average_Speed_(cm/s)	-2.539	-1.444
Run_Maximum_Variation_(%)	0.076	0.002
RF_PrintLength_(cm)_Mean	25.765	18.217
RF_SwingSpeed_(cm/s)_Mean	-0.404	-0.154
RF_StrideLength_(cm)_Mean	0.336	1.228
RH_MaxContactArea_(cm²)_Mean	-1.301	-13.419
RH_SingleStance_(s)_Mean	14.890	10.712
RH_BodySpeedVariation_(%)_Mean	0.119	0.023
LF_SingleStance_(s)_Mean	-12.533	-24.452
LF_BodySpeed_(cm/s)_Mean	-1.696	-0.628
LH_SwingSpeed_(cm/s)_Mean	0.041	0.071
OtherStatistics_Maximum_Variation_(%)	-0.006	-0.003
OtherStatistics_Cadence	2.537	2.080
PhaseDispersions_LF->RH_CStat_R	-5.049	-1.053

c d e

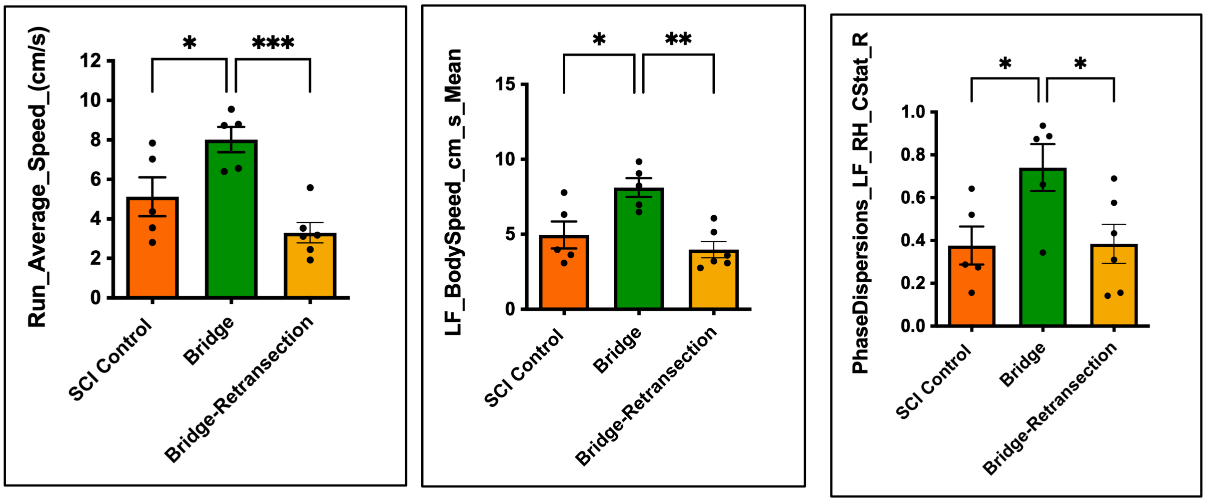

Supplemental Figure 7: Catwalk kinematic analysis at 12 weeks post-implant confirms that bridge implantation promotes motor recovery, whereas bridge transection compromises this recovery. Unbiased feature selection GBM method resulted in fifteen Catwalk variables which were utilized in a subsequent LD analysis to define the linear boundaries of the treatment groups. **(a)** LDA analysis clearly shows animals with bridge implantation separate from the other groups, and that bridge re-transection is shifted towards the SCI control group, indicating loss of recovery. Each data point represents a single mouse. **(b)** List of LD weights for each Catwalk variable identified by unbiased feature selection. **(c-e)** Representative graphs demonstrate locomotor outcomes. Data expressed as mean ± SEM (n = 5-6 per group). Groups in Figure **(c-e)** were compared using one-way ANOVA (*P≤0.05, **P≤0.01, and ***P≤0.001). LF: left forelimb; RF: right forelimb; LH: left hindlimb; RH: right hindlimb; **GBM:** Gradient Boosting Machine; **LDA:** Linear Discriminant Analysis; **LD1:** Linear Discriminant 1; **LD2:**Linear Discriminant 2.

Supplemental Table 1. List of primary antibodies, sources, and dilutions.

Primary Antibody	Host	Dilution	Manufacturer	Specificity
Olig2	Rabbit	1:250	Abcam	Oligodendrocytes
GFAP (in vitro)	Rabbit	1:3000	Dako	Astrocytes
Tubulin βIII	Mouse	1:1000	Biolegend	Neuron
STEM121	Mouse	1:500	Takara	Human Neural Stem Cell
DCX	Goat	1:500	Santa Cruz Biotechnology	Immature neurons
NeuN	Rabbit	1:500	Millipore	Mature neurons
GFAP (in vivo)	Goat	1:1000	Abcam	Astrocytes
NF-H	Rabbit	1:500	Millipore	Axons and processes
MBP	Mouse	1:250	R&D Systems	Myelin basic protein
P0	Chicken	1:500	Aves	Schwann cell myelin
GFP	Goat	1:250	Abcam	Green fluorescent protein (CST and PRV)
RFP	Rabbit	1:500	Rockland	Red fluorescent protein (mT-mNSC)
ChAT	Rabbit	1:200	Abcam	Motor neurons
CGRP	Rabbit	1:200	Sigma	Sensory fibers

CD45	Rat	1:100	Biolegend	Hematopoietic cells
CD11b	Rat	1:100	BD Biosciences	Myeloid Cells
Ly6G	Rat	1:100	Biolegend	Neutrophils (PMN)
CD68	Rat	1:100	Biolegend	Macrophages
F4/80	Rat	1:100	SeroTec	Macrophages
