# Supplementary material for: Multichannel bridges and NSC synergize to enhance axon regeneration, myelination, synaptic reconnection, and recovery after SCI
Source: NPJ Regen Med. 2024 Mar 18;9:12. doi: 10.1038/s41536-024-00356-0 (PMC10948859; doi:10.1038/s41536-024-00356-0)
Supplement: Supplementary file 2 — Reporting Summary [file 41536_2024_356_MOESM2_ESM.pdf]

nature portfolio

Corresponding author(s): Aileen J. Anderson and Usha Nekanti

Last updated by author(s): 02/05/2024

Reporting Summary

Nature Portfolio wishes to improve the reproducibility of the work that we publish. This form provides structure for consistency and transparency in reporting. For further information on Nature Portfolio policies, see our Editorial Policies and the Editorial Policy Checklist.

Statistics

For all statistical analyses, confirm that the following items are present in the figure legend, table legend, main text, or Methods section.

n/a	Confirmed	
☐	☒	The exact sample size (*n*) for each experimental group/condition, given as a discrete number and unit of measurement
☐	☒	A statement on whether measurements were taken from distinct samples or whether the same sample was measured repeatedly
☐	☒	The statistical test(s) used AND whether they are one- or two-sided<br>*Only common tests should be described solely by name; describe more complex techniques in the Methods section.*
☐	☒	A description of all covariates tested
☐	☒	A description of any assumptions or corrections, such as tests of normality and adjustment for multiple comparisons
☐	☒	A full description of the statistical parameters including central tendency (e.g. means) or other basic estimates (e.g. regression coefficient) AND variation (e.g. standard deviation) or associated estimates of uncertainty (e.g. confidence intervals)
☐	☒	For null hypothesis testing, the test statistic (e.g. *F*, *t*, *r*) with confidence intervals, effect sizes, degrees of freedom and *P* value noted<br>*Give P values as exact values whenever suitable.*
☒	☐	For Bayesian analysis, information on the choice of priors and Markov chain Monte Carlo settings
☒	☐	For hierarchical and complex designs, identification of the appropriate level for tests and full reporting of outcomes
☒	☐	Estimates of effect sizes (e.g. Cohen's *d*, Pearson's *r*), indicating how they were calculated

Our web collection on statistics for biologists contains articles on many of the points above.

Software and code

Policy information about availability of computer code

Data collection	All details regarding data collection are described in the Method section at the relevant paragraph. Flow cytometric data was acquired using a BD FACS Aria II. Image acquisition was performed using a ZEISS Axio Imager II light microscope and ZEISS LSM 900 with Airyscan 2 microscope. Locomotor recovery tasks were performed using a horizontal ladder beam and Noldus CatWalk XT system.
Data analysis	All softwares used for analysis are described in the relevant Methods section. Flow cytometric data was analyzed using the FlowJo software 10.8.2. For in vitro hNSC fate, Imaris software 7.5.2 was used. Imaris software 7.5.2 was used to determine hNSC fate in vitro. MicroBrightField Stereo Investigator version 2020.1.3 software was used to quantify hNSC fate in vivo, and Imaris software 9.6.0 was used to quantify neurofilament and PRV-GFP filament volume. CatWalk runs were auto-classified using the CatWalk software version 10.1, and CatWalk analysis was conducted in R package . GraphPad Prism (version 9.2.0) was used for all statistical analysis.

For manuscripts utilizing custom algorithms or software that are central to the research but not yet described in published literature, software must be made available to editors and reviewers. We strongly encourage code deposition in a community repository (e.g. GitHub). See the Nature Portfolio guidelines for submitting code & software for further information.

Data

Policy information about availability of data

All manuscripts must include a data availability statement. This statement should provide the following information, where applicable:

- Accession codes, unique identifiers, or web links for publicly available datasets
- A description of any restrictions on data availability
- For clinical datasets or third party data, please ensure that the statement adheres to our policy

The main data supporting the results of this study are available within the paper and its Supplementary Information. The raw and analyzed datasets generated during the study are too large to be publicly shared, but they are available from the corresponding author upon reasonable request.

Human research participants

Policy information about studies involving human research participants and Sex and Gender in Research.

Reporting on sex and gender	No human participants were involved in this study.
Population characteristics	No human participants were involved in this study.
Recruitment	No human participants were involved in this study.
Ethics oversight	No human participants were involved in this study.

Note that full information on the approval of the study protocol must also be provided in the manuscript.

Field-specific reporting

Please select the one below that is the best fit for your research. If you are not sure, read the appropriate sections before making your selection.

☒ Life sciences ☐ Behavioural & social sciences ☐ Ecological, evolutionary & environmental sciences

For a reference copy of the document with all sections, see nature.com/documents/nr-reporting-summary-flat.pdf

Life sciences study design

All studies must disclose on these points even when the disclosure is negative.

Sample size

No statistical methods were used to predetermine sample sizes specifically for this manuscript; we relied on historical calculations for power analysis for this injury model and the behavioral tasks planned. Calculation of power analysis for histological measures was not possible, as these analyses have not been done quantitatively previously in this model. However, all flow cytometric, anatomical, and behavioral experiments were performed using multiple animals, and sample size is detailed in the method section.

Data exclusions

Data exclusion criteria for animal behavior studies are described in the method section. Tissue sections were excluded from histological analysis if any part of the tissue or PLG bridges material was damaged or lost during dissection, sectioning, or staining procedures. In addition, mouse with anatomical defects, non-specific surgical damage, or transplantation errors was excluded from both histological and behavior analysis.

Flow cytometric analysis was carried out on C57BL/6 mice. Each group comprised of 5 mice. However, in the SCI control group, one mouse from the 1DPI time point and one mouse from the 8WPI time point were excluded due to the failure of the myelin depletion procedure and poor staining outcomes. Additionally, one mouse from the 24 WPI time point was excluded because it was identified as an outlier by Grubbs' test. Therefore, the total number of mice in these groups was reduced to 4. PLG bridge groups comprised n=5 mice in all groups except at 1WPI time point, where a mouse died post-op, resulting in n=4 at that time point.

For in vitro analysis of hNSC fate, each group had n=4 biological replicates. In the PMN CM-PLG scaffold group, there was one lost data point for oligodendrocyte fate. This was caused by improper placement of the PLG scaffold within the cell culture plate well, resulting in media evaporation and cell death on the scaffold.

Replication

Flow cytometric, histological, and behavioral experiments included independent biological replicates. The improvement of motor recovery in the groups that received the PLG bridge alone and hNSC alone was consistent with our previous findings. Furthermore, we observed parallel flow cytometric analysis of the innate immune cell profile following SCI control or bridge implantation in both C57BL/6 mice and Rag1-deficient mice (Fig. 2 and Supplemental Fig. 2). hNSC fate selection in vitro in the presence of a PLG bridge and inflammatory cues was replicated in at least three biological replicates.

Randomization

All experiments, including animal care, behavior data acquisition and analysis, histological analysis, pseudorabies virus (PRV) tracing, and flow cytometric analysis, were conducted by researchers who were blinded to the study groups (masking), and random group assignment was

	utilized.
Blinding	Behavior data acquisition and analysis, image acquisition and histological analysis, flow cytometric analysis, and in vitro hNSC fate analysis were performed by investigators blinded to the experimental groups. A single individual in the laboratory held the study code.

Reporting for specific materials, systems and methods

We require information from authors about some types of materials, experimental systems and methods used in many studies. Here, indicate whether each material, system or method listed is relevant to your study. If you are not sure if a list item applies to your research, read the appropriate section before selecting a response.

Materials & experimental systems

n/a	Involved in the study
☐	☒ Antibodies
☐	☒ Eukaryotic cell lines
☒	☐ Palaeontology and archaeology
☐	☒ Animals and other organisms
☒	☐ Clinical data
☒	☐ Dual use research of concern

Methods

n/a	Involved in the study
☒	☐ ChIP-seq
☐	☒ Flow cytometry
☒	☐ MRI-based neuroimaging

Antibodies

Antibodies used	All antibody sources and dilutions utilized in this study are listed in Supplemental Table 1.
Validation	Antibodies were validated by using a secondary-only control and confirming the lack of fluorescent signal for each antibody. Antibodies were also validated based on the morphological and spatial distribution of labeling, which were similar to previously published images and the manufacturer's example images. Additional validation details can be found on the manufacturer's website.

Eukaryotic cell lines

Policy information about cell lines and Sex and Gender in Research

Cell line source(s)	Multipotent human neural stem cell line, UCI161 (referred as hNSC) was established in the Anderson laboratory at UCI from fetal brain tissue procured at gestational 16 weeks under UCI hSCRO and IRB approval. These cells are were derived prior to June 5, 2019 (NIH NOT-OD-19-128)<br><br>mT-mNSC line was established in our lab at UCI from cortices of 24Gt(ROSA)26Sortm4(ACTB-tdTomato-EGFP)Luo/J mouse (JAX mice #007576, The Jackson Laboratory, Bar Harbor, ME) at embryonic day 11.5 (E11.5)
Authentication	UCI161 hNSC have been used in multiple studies in our laboratory, including in previous publications (Benavente, F., et al. 2020). Additionally, all cell lines were treated with neural differentiation media for 14 days in vitro and immunostained with neural fate markers to verify retention of multilineage potential, and all cell lines in our laboratory are banked for use at low passage number (<15), and also routinely screened for normal karoytype. mT-mNSC membrane-targeted tdTomato reporter expression was previously published (Powers, B.E., et al. 2013), and validated observationally in these cells in vitro in our laboratory. hNSC were further validated for normal karyotype maintenance, and to be negative for adventitious agents.
Mycoplasma contamination	All cell lines were routinely tested for mycoplasma contamination. The cell lines used in this study were verified to be mycoplasma-free before using them in any in vitro and in vivo experiments.
Commonly misidentified lines<br>(See ICLAC register)	None used

Animals and other research organisms

Policy information about studies involving animals; ARRIVE guidelines recommended for reporting animal research, and Sex and Gender in Research

Laboratory animals	Profiling of the innate inflammatory microenvironment following SCI was performed using C57BL/6 mice (8 to 10 weeks at the time of injury; JAXmice # 000664) and Rag1 mice (8 to 12 weeks at the time of injury; JAXmice # 002216). hNSC transplantation studies were conducted in immunodeficient Rag1 mice (aged 11 to 19 weeks at the time of injury). Multipotent cell membrane-localized tdTomato mouse neural stem cell (mT-mNSC) transplantation studies were conducted in Crym-ZsGreen1 transgenic mice (9 to 15 weeks at the time of injury). PLG bridge retransection study is performed in transgenic EMX1:ROSA-CRYM-RFP mice. All mice were group housed with 2-4 cage mates.
Wild animals	No wild animals were used in this study

Reporting on sex	To prevent the occurrence of bladder complications and urolithiasis, which are common in male mice subsequent to SCI, female mice were utilized in this study.
Field-collected samples	The study did not involve samples collected from the field.
Ethics oversight	Animal care, behavior acquisition, and data analysis were performed by investigators blinded to the experimental groups. All animal housing conditions, procedures, and animal care were approved by the UCI Institutional Animal Care and Use Committee (IACUC). Derivation and usage of human neural stem cell line UCI 161 for all in vitro and in vivo work was reviewed and approved by the UCI human Stem Cell Research Oversight Committee (hSCRO).

Note that full information on the approval of the study protocol must also be provided in the manuscript.

Flow Cytometry

Plots

Confirm that:

☒ The axis labels state the marker and fluorochrome used (e.g. CD4-FITC).

☒ The axis scales are clearly visible. Include numbers along axes only for bottom left plot of group (a 'group' is an analysis of identical markers).

☒ All plots are contour plots with outliers or pseudocolor plots.

☒ A numerical value for number of cells or percentage (with statistics) is provided.

Methodology

Sample preparation	Innate immune cell time course profiling following SCI was performed by dissecting C4-C6 spinal segment at 1 day post-injury (DPI), 1 week post-injury (WPI), 4WPI, 8WPI or 24WPI. Spinal cord tissue was dissociated using mechanical and enzymatic methods as previously described. Myelin debris was removed using Myelin Removal Magnetic Beads II kit (Miltenyi Biotec, Auburn, CA) using an auto-MACS Pro Separator according to manufacturer instructions (Miltenyi Biotec, Auburn, CA). Cells collected after myelin removal were suspended in 0.85% ammonium chloride (diluted in sterile water) for five minutes to lyse red blood cells. The cell suspension was resuspended in PBS supplemented with 2% FBS (Thermo Fisher Scientific, Waltham, MA). Then the cells were stained with 7AAD viability dye (Thermo Fisher Scientific, Waltham, MA), CD45, CD11b, Ly6G, and CD68 antibodies.
Instrument	The data was acquired using a BD FACS Aria II Flow cytometer
Software	Data analysis was performed using FlowJo software version10.8.2
Cell population abundance	To gate for CD45+CD11+ myeloid events, a minimum of 10,000 live events were analyzed. As shown in Figure 2A, these total myeloid cells were further gated to determine the proportion of Ly6G+, Polymorphonuclear leukocytes (PMN), and CD68+ macrophages (MØ)/microglia.
Gating strategy	Figure exemplifying the gating strategy is provided in the supplemental figure 1.<br>To ensure the detection of a true positive signal for each fluorescence channel, we used single stain compensation controls and fluorescence minus one (FMO) controls to draw the gates. For analysis, FSC-A vs SSC-A scatter was used to gate for bulk cell population, FSC-A vs. FSC-H was applied to minimize doublet selection. Next, 7AAD negative live cells were gated to analyze the number of CD45+CD11b+ myeloid events. Total myeloid cells were further gated to analyze the proportion of Ly6G+ PMN (e), and CD68+ MØ/microglia subpopulations.

☒ Tick this box to confirm that a figure exemplifying the gating strategy is provided in the Supplementary Information.
